# Codon usage is associated with the evolutionary age of genes in metazoan genomes

**DOI:** 10.1186/1471-2148-9-285

**Published:** 2009-12-08

**Authors:** Yosef Prat, Menachem Fromer, Nathan Linial, Michal Linial

**Affiliations:** 1 School of Computer Science and Engineering, The Hebrew University of Jerusalem, Jerusalem, 91904, Israel; 2 Sudarsky Center for Computational Biology, The Hebrew University of Jerusalem, Jerusalem, 91904, Israel; 3 Deptartment of Biological Chemistry, Institute of Life Sciences, The Hebrew University of Jerusalem, Jerusalem, 91904, Israel

## Abstract

**Background:**

Codon usage may vary significantly between different organisms and between genes within the same organism. Several evolutionary processes have been postulated to be the predominant determinants of codon usage: selection, mutation, and genetic drift. However, the relative contribution of each of these factors in different species remains debatable. The availability of complete genomes for tens of multicellular organisms provides an opportunity to inspect the relationship between codon usage and the evolutionary age of genes.

**Results:**

We assign an evolutionary age to a gene based on the relative positions of its identified homologues in a standard phylogenetic tree. This yields a classification of all genes in a genome to several evolutionary age classes. The present study starts from the observation that each age class of genes has a unique codon usage and proceeds to provide a quantitative analysis of the codon usage in these classes. This observation is made for the genomes of *Homo sapiens*, *Mus musculus*, and *Drosophila melanogaster*. It is even more remarkable that the differences between codon usages in different age groups exhibit similar and consistent behavior in various organisms. While we find that GC content and gene length are also associated with the evolutionary age of genes, they can provide only a partial explanation for the observed codon usage.

**Conclusion:**

While factors such as GC content, mutational bias, and selection shape the codon usage in a genome, the evolutionary history of an organism over hundreds of millions of years is an overlooked property that is strongly linked to GC content, protein length, and, even more significantly, to the codon usage of metazoan genomes.

## Background

The degeneracy of the genetic code implies that different codon triplets encode the same amino acid. The frequencies with which these different codons are used vary significantly between different organisms and between proteins within the same organism [1].

Many studies have analyzed the differences in codon usage across species [2,3]. Some of the main conclusions of these studies are: (i) In prokaryotes, archaea, and single-cell eukaryotes [4], translational efficiency (or fidelity) underlies the strong codon usage bias discovered for highly expressed genes [5]. This correlation is valid as well in multicellular organisms, such as worms [6,7], flies, and plants [8,9], but does not hold in higher multicellular organisms [10]; (ii) There exists a strong correlation between codon usage and genomic GC content. This result was demonstrated in diverse organisms ranging from bacteria to metazoa. Moreover, it was even suggested that human codon usage is determined solely by GC content and its isochores composition [1]. The causal relationships between GC content, codon usage, and the underlying evolutionary constraints that may have shaped them are still not fully understood.

Nonetheless, several evolutionary processes have been postulated as the major factors that determine codon usage: selection, mutation, and genetic drift. However, the relative contribution of each of these factors in different species remains debatable [11-14]. For a number of different organisms, it was suggested that codon usage is best explained by selection for tRNA abundance, gene expression levels, and translational optimization [15]. In other cases, the dominant roles were attributed to mutation bias for local composition, mutation rate, mutation preference [16], biased gene conversion, and recombination rates [17]. Among other attributes considered are gene and protein properties [18], including protein structure [19], gene length [9], and mRNA characteristics (e.g., secondary structure) [20]. Mutation bias towards the transcribed strand [21], environmental conditions [22], and generation time [23] were also proposed in explaining the preferred usage of codons in specific genes and some genomes.

The availability of a substantial number of complete metazoan genomes provides an opportunity to inspect the codon usage signal vis-a-vis the age of the genes that contain these codons. Here, we examine the varying use of codons in different groups of genes, where the groups are defined according to their relative evolutionary age within a single organism. We show a significant coupling between the evolutionary age of a gene and its codon preferences in representative metazoan genomes. We also show that the GC content of a gene and its length are associated with its evolutionary age. However, we demonstrate that the latter two linkages provide only a partial explanation of the codon usage bias. We propose that the evolutionary history of genes has been maintained in the frequencies of their codons throughout extremely long evolutionary processes.

## Results and Discussion

The analysis of metazoan codon usage is made possible by the availability of a large number of complete genomes. Hundreds of eukaryotic genomes are currently at their final stages of assembly and genome annotation. Complete high-quality proteomes of about 40 animal genomes are available as well. These resources have allowed us to determine with much certainty, for each of the protein sequences of a given organism, if homologues are present or absent along the evolutionary phylogenetic tree.

### Codon usage and evolutionary age

Proteins encoding gene sequences were obtained from the ENSEMBL database [24]. To avoid bias due to genome annotation quality, we focused our analysis solely on genes marked as 'known'. Very short genes were also removed to avoid statistical bias in all subsequent homology searches (see Methods).

**Figure 1:**
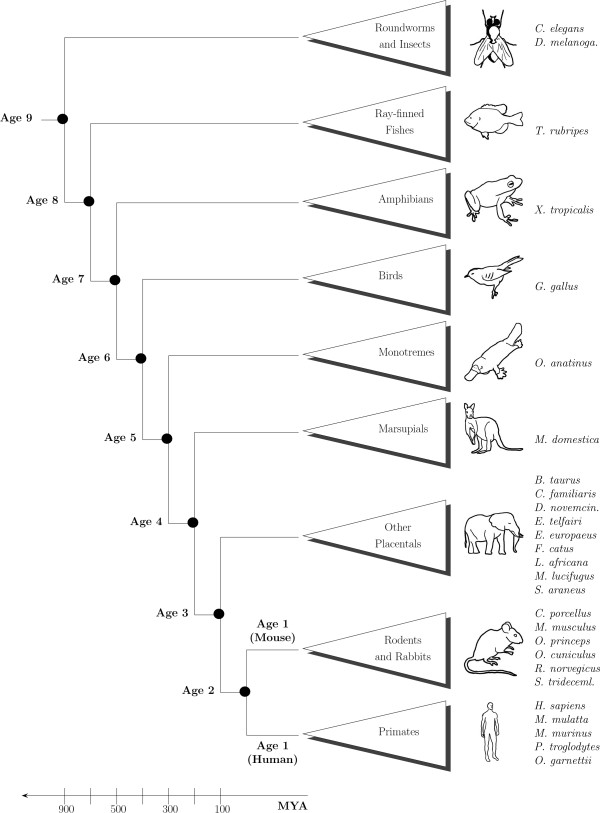
**Phylogenetic tree used to define the relative age groups for the human and mouse genes**. The labeled age classes were defined as the major evolutionary branching points with respect to the 27 genomes analyzed and the species of interest (human or mouse). Thus, genes are grouped according to their estimated time of appearance in evolution. For example, human genes in Age group 5 are presumed to have appeared after the split between birds and mammals, since they do not have homologues in the non-mammal species studied. On the other hand, they already existed in the least common ancestor (LCA) of all mammals, as evidenced by their respective homologues in *O. anatinus*. The species included in the analysis are: *C. elegans *(worm), *D. melanogaster *(fruitfly), *T. rubripes *(fugu), *X. tropicalis *(xenopus), *G. gallus *(chicken), *O. anatinus *(platypus), *M. domestica *(Gray Short-tailed opossum), *B. Taurus *(cow), *C. familiaris *(dog), *D. novemcinctus *(nine-banded armadillo), *E. telfairi *(lesser hedgehog tenrec), *E. europaeus *(west european hedgehog), *F. catus *(cat), *L. Africana *(elephant), *M. lucifugus *(bat), *S. araneus *(common shrew), *C. porcellus *(guinea pig), *M. musculus *(mouse), *O. princes *(pika), *O. cuniculus *(rabbit), *R. norvegicos *(rat), *S. tridecemlineatu *(squirrel), *P. troglodytes *(chimpanzee), *M. mulatta *(macaque), *M. murinus *(gray mouse lemur), *O. garnettii *(bushbaby), and *H. sapiens *(human). For the analysis of the human genome, Age 1 includes only primate-specific genes, while for the analysis of the mouse genome, Age 1 includes only rabbit and rodent-specific genes. Note that the evolutionary time scale (in millions of years ago, MYA) is approximate.

We divided the set of about 17,000 analyzed human genes into 9 groups according to the evolutionary age of each gene (Table 1). The age of a gene was determined by the evolutionarily most distant genome containing an identified homologue to that gene. Relative evolutionary distance was based on the accepted phylogenetic tree (Figure 1). Homology relationships were extracted from ENSEMBL and are based on an exhaustive list of 27 fully sequenced genome annotations that represent the main model organisms of the animal kingdom (Figure 1). Note that despite the large variation in group sizes (Tables 1 and 2), even the smallest age groups contain more than enough codon appearances to make statistically robust conclusions (e.g., human Age group 2 contains over 42,000 codon counts).

**Table 1:** Partition of *H. sapiens *and *M. musculus *genes into age groups

	Age group*	# Genes	Coding region GC content		Protein length	
			
			Mean	SD	Mean	SD
	1	612	0.54	± 0.10	363	± 466
	2	97	0.53	± 0.11	441	± 352
	3	1202	0.54	± 0.10	518	± 504
	4	581	0.55	± 0.09	531	± 472
Human	5	648	0.52	± 0.09	477	± 424
	6	592	0.51	± 0.09	681	± 686
	7	605	0.53	± 0.10	571	± 400
	8	3914	0.54	± 0.09	557	± 498
	9	9023	0.53	± 0.09	525	± 399
	
	Total	17274				

	1	983	0.49	± 0.10	384	± 391
	2	39	0.51	± 0.07	590	± 446
	3	861	0.51	± 0.07	534	± 516
	4	854	0.50	± 0.08	464	± 428
Mouse	5	908	0.48	± 0.07	414	± 362
	6	708	0.50	± 0.07	623	± 659
	7	645	0.52	± 0.07	555	± 409
	8	4119	0.54	± 0.07	546	± 458
	9	9099	0.53	± 0.06	526	± 396
	
	Total	18216				

*Ordinal evolutionary age, relative to the reference species under analysis

Partition of all genes in *H. sapiens *and *M. musculus *into 9 non-overlapping age groups along the evolutionary tree. For each age group, the number of genes it contains, its average GC content (with standard deviation), and the average length of the proteins (number of amino acids/codons analyzed) that its genes encode (with standard deviation) are listed. The age groups are as in Figure 1.

**Table 2:** Partition of *D. melanogaster *genes into age groups

a					
**D. melanogater gene is in Age group:**	**If the furthest species containing a homologue is among:**				

1	***D. melanogaster***, *D. sechellia*, *D. simulans*, *D. erecta*, *D. yakuba*				
2	*D. ananassae*				
3	*D. pseudoobscura*, *D. persimilis*				
4	*D. wilistoni*				
5	*D. mojavensis*, *D. grimshawi*, *D. virilis*				
6	*Ades aegypti., Anopheles gambiae*.				
7	Vertebrates, *C. elegans*				
8	*Saccharomyces cerevisiae*				

**b**					

**Age group**	**# genes**	**Coding region GC content**		**Protein length**	
		
		**Mean**	**SD**	**Mean**	**SD**

1	336	0.49	± 0.06	412	± 892
2	130	0.49	± 0.06	401	± 406
3	160	0.49	± 0.06	394	± 245
4	147	0.51	± 0.06	456	± 421
5	2582	0.54	± 0.06	568	± 691
6	1841	0.55	± 0.05	650	± 625
7	3155	0.55	± 0.04	578	± 471
8	2632	0.55	± 0.04	543	± 406

Total	10983				

Partition of all genes in *D. melanogaster *into 8 non-overlapping age groups along the evolutionary tree. (a) Genes were divided into 8 age groups. The 6 youngest groups (1 to 6) are a "high-resolution" partition, enabled by the recent sequencing of 12 members of the Drosophilidae family and 2 mosquito species. (b) For each age group, the number of genes it contains, its average GC content (with standard deviation), and the average length of proteins (number of amino acids/codons analyzed) that its genes encode (with standard deviation) are listed.

For each group of genes, codon usage frequencies were independently calculated for each of the amino acids. Thus, each of the 59 redundant codons that account for these 18 amino acids were assigned a number between 0 and 1 (see Methods).

**Figure 2:**
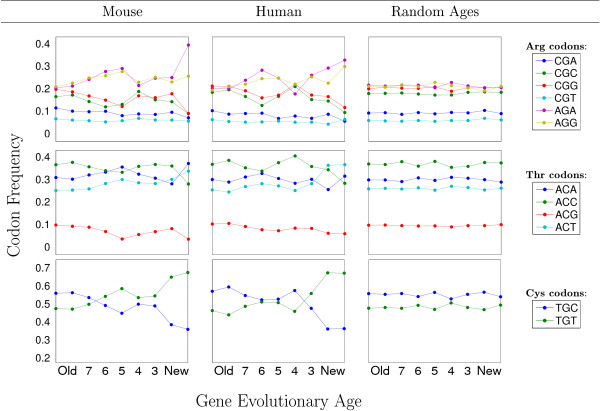
**Age-dependent codon usages for representative codons**. The age dependent codon usages for the arginine (top), threonine (middle), and cysteine (bottom) codons for the mouse and human genes are shown. In the right column, the codon usages for these amino acids after a random reshuffling of the age assignments for the human genes are shown. See Figure 1 for the definition of the age groups used.

The analysis of the 9 evolutionary age groups reveals substantial differences in their codon usage. This was observed for almost all codons of all amino acids. Representative results for arginine, threonine, and cysteine are depicted in Figure 2 (middle column). As a comparison, we show the codon usage variation for the ~17,000 genes within 9 randomly assigned age groups of similar sizes (Figure 2, right column). It is evident that the randomized grouping results in a complete loss of age dependency.

To robustly test the statistical significance of our observation for each of the 59 analyzed codons, we measured the variance of the codon usage between the 9 age groups. This variance was compared to that of 9 randomly selected gene groups with similar sizes, and this comparison was independently repeated 10,000 times. The variance of the codon usage among the 9 age groups was greater than the random groups' variance in more than 95% of the tests for 58 codons (p < 0.05), and 94% for the CTA codon (encoding leucine, p < 0.06). These tests confirm the observation that the age groups are significantly different from one another with respect to their codon usage. Another noteworthy observation is that a number of codon frequencies change monotonically with the age of the gene group considered (Additional file 1).

Recent research of codon usage bias has typically employed measures such as the codon adaptation index (CAI) [25] or the effective number of codons (ENC) [26]. However, since these measures are gene-focused, they were not appropriate for this study, where we characterized hundreds of genes at a time, without the use of a reference set or other simplifying assumptions. In addition, other studies have used relative synonymous codon usage (RSCU) values to measure the deviation from random per-amino acid codon usage [27]. We did not use this measure here since we were interested in measuring the variation of the frequency for each specific codon between different age groups, without comparing between codons of the same amino acid (see discussion in [28,29]).

**Figure 3:**
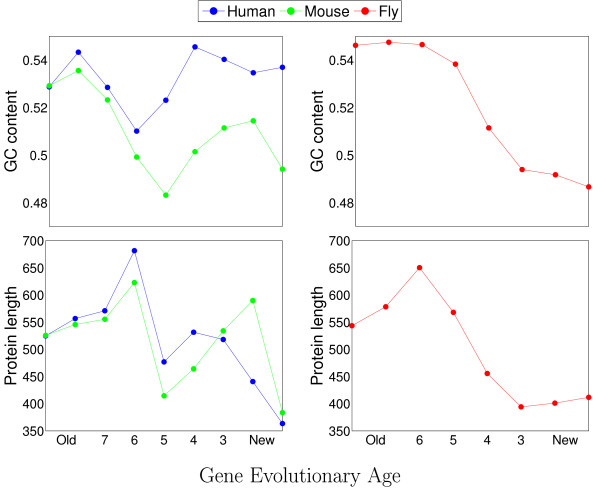
**Age-dependent GC content and length of human, mouse, and fly genes**. For each age group, the average GC content of the coding regions of the genes, or average protein length, is shown. See Figure 1 and Table 2 for the definition of the age groups used. For each of human, mouse, and fly, the variance between age groups for both GC content and protein length is statistically significant (permutation test, p < 10^-6^).

Previous studies have proposed that GC content is a major determinant of codon usage [30]. We thus examined the GC content differences between the age groups. Indeed, the GC content within the coding regions of the ~17,000 human genes shows significant variance between the 9 groups (Figure 3) and is more variable than in randomly selected groups (permutation test, p < 10^-6^). Thus, we report here a seemingly overlooked association between the GC content and the evolutionary age of a gene. Previous studies have shown a decrease in GC-rich isochores in mammalian genomes [31], as can also be noted among synonymous codons from the newest age groups (Additional files 1 and 2).

Using similar tests, we found that protein length, which has also been suggested to correlate with codon usage [9] is associated (p < 10^-6^) with the age group to which the gene belongs (Figure 3).

**Figure 4:**
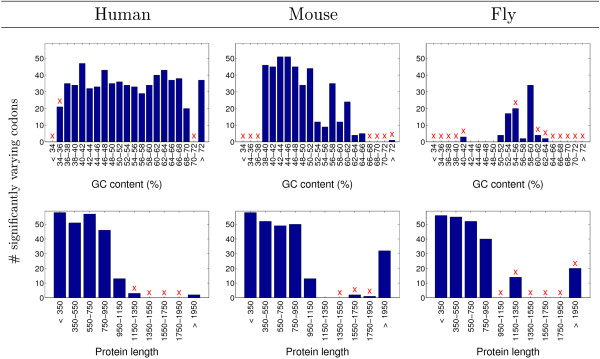
**Age-dependent codon usage for fixed GC content and length**. For each of human, mouse, and fly (left to right), its genes were binned by either their GC contents (top) or lengths (bottom). For each such bin, the number of codons (out of the 59 analyzed) with statistically significant age-dependent variance is shown. A particular codon was labeled as being age-dependent if its variance between ages was different than random (false discovery rate (FDR) corrected for multiple hypotheses < 0.05). Red 'X' mark bins for which the sub-division into age groups resulted in some groups having fewer than 5 genes; the results for such bins should be disregarded, since they are statistically inadequate.

In order to uncouple the age dependence of GC content and gene length from that of codon usage, we tested whether genes with very similar GC content (or length) still show a significant linkage between gene age and codon usage. We thus binned the genes into sets of similar GC content (or length) and further divided each such set into the 9 age groups defined above. For each GC content (or length), the variance among the age groups was re-tested. We found statistically significant variation between the age groups for many of the codons (Figure 4). Thus, the coupling between age and GC content (or age and protein length) does not entirely explain our main observation indicating age-dependent codon usage (Figure 2, middle column).

To additionally confirm our conclusion that GC content does not dominate the age-dependent codon usages observed, we performed the following novel information-theoretic test. Intuitively, if changes in GC content were the only factor in causing aberrations from the overall codon usage of the organism, we would expect the codon usage of a particular age group to be dictated by its GC content, while deviating as little as possible from the overall codon usage of the genome. Thus, for each group, we determined the codon usage expected to comply with its GC content. In detail, we calculated the usage that minimizes the Kullback-Leibler divergence (D_KL_) with the genomic codon usage, while constraining the GC content (see Methods). We found the observed codon usages for each of the age groups to be significantly different than those expected based on GC alone (χ^2 ^test, p < 10^-10^); this result was consistent for human, mouse, and fly (see below).

### Codon usage preference by evolutionary age is a universal phenomenon

We tested whether the association between evolutionary age and codon usage preferences observed in *Homo sapiens *carries over to other metazoa. To this end, we applied a similar analysis for the *Mus musculus *and the *Drosophila melangoaster *genomes (Table 2). For the latter genome, we overcome the uneven full-genome sampling of the evolutionary tree from human to fly by taking advantage of the recent sequencing and annotations efforts for 12 species from the Drosophila genus [32].

For the mouse and fly species, a linkage between gene age and codon usage biases (Figure 2, left column and Additional files 2 &3) was confirmed by applying the same tests applied for the human genome. The variance in codon usage was significantly higher than random (p < 0.05) for 54 codons in mouse (excluding: CGT (R), CTC (L), CCT (P), GCC (A), GTC (V)), and for 57 codons in fly (the exceptions being CGT (R) and GTC (V)). Therefore, the linkage between codon usage and gene age is not specific to the human genome and is likely to apply to other metazoans as well.

For the fly genome, the correlation between gene age and GC content seems to be stronger than for the human and mouse genes (Figure 3). And, indeed, while the GC content only weakly explains the age dependency of codon usage for mouse genes (similar to that observed for human), the age-related codon bias of fly genes seems to be mostly, but not entirely, dominated by the GC content (Figure 4, middle-top and right-top panels, respectively). It is also worth noting that, in *D. melanogaster*, age-dependent monotonic behavior was observed in the GC content as well as in the codon usage for most of the 59 codons (Additional file 3), perhaps implying a stronger link between these two age-dependent phenomena. Furthermore, for almost all amino acids in the fly, the usage of synonymous codons is more uniform in the newer age groups relative to the old ones (Additional file 3). This may reflect the combination of: (i) the GC content necessarily restricts the possible uniformity of codon usage [33] (Figure 3, old age groups); (ii) a potential overlap between the old age groups and slow evolving genes, which have previously been shown to possess large differences in the usage of synonymous codons [34].

We now set out to test whether the coupling found between the codon usage and gene age behaves similarly in human, mouse, and fly. For simplicity, we grouped the genes of each organism into two groups representing 'new' and 'old' genes. The 'new' set contains all genes that are primate-specific (for human), rodent and rabbit-specific (for mouse), and melanogaster subgroup-specific (for fly). The 'old' ones are those that are not included in the 'new' group (see Figure 1 and Table 2).

We then measured the deviation of the codon usage for the 'new' genes from that of the 'old' genes, for each of the three model organisms. The codon usage was represented by a 59-coordinate vector (one coordinate for each codon, whose value is its relative usage frequency among the codons encoding its amino acid). Hence, the deviation between the 'new' and 'old' groups in each genome is quantified as the difference of the 'new' and 'old' vectors (see Methods). Thus, these calculations yield three vectors, each representing the influence of age on the codon usage in each organism. When we measure the angle between the three vectors in the 59-dimensional space, a remarkable resemblance is observed. These deviation vectors have practically the same directions (i.e., multidimensional "trajectory") for the human, mouse, and fly, with a statistical significance of p < 10^-8 ^(Table 3, see Methods). This is particularly noteworthy, since the 'new' groups are expected to have evolved independently, since, by definition, they include genes that appeared after the separation of each pair among human, mouse, and fly.

**Table 3:** Pairwise comparison of age-dependent codon usage deviation vectors for human, mouse, and fly genes

	*θ*	cos(*θ*)	p-value
*θ*(human, mouse)	18.3°	0.9494	3.6 × 10^-12^
*θ*(human, fly)	37.0°	0.7984	8.5 × 10^-9^
*θ*(mouse, fly)	29.3°	0.8719	2.3 × 10^-10^

For each of the three genomes analyzed, the deviation vector was defined as the difference of the codon usage vectors for 'old' and 'new' genes. The angle between each pair of 59-dimensional deviation vectors was measured (*θ*) and the p-value of obtaining this angle was calculated (see Methods).

**Figure 5:**
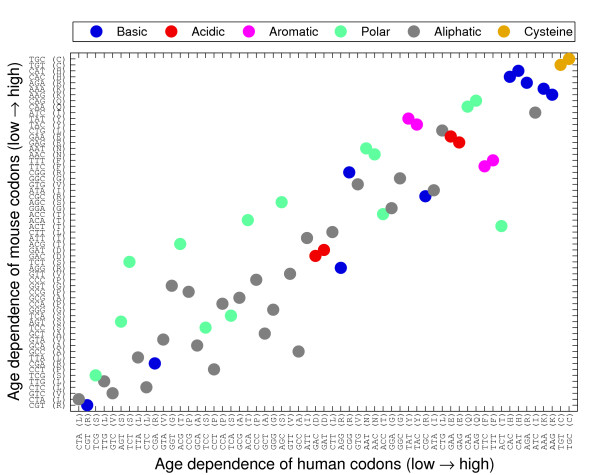
**Codon age-responsiveness for the 59 degenerately coding codons**. For each codon, the age-dependent variance was calculated. For each genome, the 59 resulting variance scores were rank-ordered. The 59 codons are sorted by their rank-ordering in the human genome, and the rank-ordering in the mouse genome is compared. A strong overall similarity of codon rank-ordering between the human and mouse genomes is shown. Spearman's rank correlation test: *ρ *= 0.92, p-value < 10^-6^. The codons are colored according to a standard biochemical grouping of the amino acids for which they encode.

We subsequently proceeded to quantify the level of dependence of the usage frequency on the age signal, for each of the 59 codons. Namely, we examine the extent to which different codons (or their amino acids) encapsulate the evolutionary age signal and ask which codons are more "sensitive" to gene age, across the three metazoan representatives examined. To this end, we ranked the codons in each organism according to their responsiveness to evolutionary age (Figure 5), using the variance among the 9 age groups. Indeed, human and mouse codons showed similar patterns regarding the specific dependency of age (Spearman's rank correlation test: *ρ *= 0.92, p < 10^-6 ^with permutations test). Moreover, this pattern was somewhat conserved for Drosophila (*ρ *= 0.74, p < 10^-6^). We tested the properties of the codons showing high responsiveness toward evolutionary age (and those that are indifferent to it) according to the biochemical grouping of the corresponding amino acids. No clear correlation was found between the biochemical grouping of the amino acids and the ranks of their codons.

### Properties of evolutionary age groups

We further checked whether the evolutionary age groupings might be correlated with some functional classification. It is well known that certain functions are found in only specific parts of the evolutionary phylogenetic tree, e.g. those involved in morphogenesis, organ development, differences in brain function, and behavior. Moreover, certain traits (e.g., the immune system and pathogen defense mechanisms) were acquired late in metazoan evolution. Indeed, studying the trend of development-specific genes along the evolutionary tree supports the notion that the molecular signal of evolutionary history is partially retained [35,36].

We tested the possible enrichment of Gene Ontology (GO) annotations [37] in each of the 9 age groups of human genes. It turns out that a few functional annotations are enriched in some age groups. Specifically, newer age groups were enriched with "nucleic acid binding" genes, while older groups showed enrichment with the process of "molecular transducer activity". The function that was most prevalent in the oldest age group was "catalytic activity". However, the number of genes annotated with these GO terms was relatively small and thus only insignificantly influenced the group character.

## Conclusion

In this study, we provide an unbiased measurement, in metazoan genomes, of the effect of the evolutionary age of genes on their codon usage. We adopt a critical statistical perspective that analyzes the codon usage signal on a genomic scale, rather than from a gene-centric point of view. This approach has revealed weak signals that may otherwise be masked by gene-to-gene variation.

Our results are quite surprising. Most correlations that were previously suggested to dominate the determination of codon usage are time-independent, thereby implying that the evolutionary history of a gene or a species is less important than its current properties. Most studies that suggest selection as the major driving force for codon bias have analyzed protein structures, mRNA stability, expression efficiency, and recombination mechanisms [17]. These studies took advantage of the availability of absolute gene expression, proteomic expression levels, and expression breadth. These features are used as appropriate approximations to selective forces. Of note, none of these models include an element of evolutionary age. Indeed, since it is assumed that current metazoan genomes are near equilibrium with respect to mutation and selection [30], it was unexpected to find age-dependent differences in the codon usage of genes, as reported here.

We have herein reported a phenomenon relating the age of gene groups and their codon preferences. The evolutionary mechanisms underlying this phenomenon are yet to be discovered. It is important to note that our assessment of evolutionary age might be influenced by biological noise, such as rapidly evolving genes. In addition, our analysis could be confounded by cases where there is no gene homologue in the representative genomes for some branches of the evolutionary tree. Thus, one should be cautious about the definition of homologues, which could lack detection sensitivity due to the uneven and somewhat biased selection of genomes that were completely sequenced. For example, an intermediate genome that is not yet sequenced can provide a missing link that will redefine the partition of an age group and will thus directly affect the assignment of a gene to its appropriate age. Mechanistically, this could be the consequence of gene loss, lateral transfer of genetic material (through retroviral dynamics), but also through recombination and gene conversion processes [17]. These potential drawbacks increase the unavoidable inaccuracy of the genomic data used in this study. Notwithstanding these reservations, we did find a significant degree of age dependency for codon usage. We have also reported here phenomena of the dependence of GC content and protein length on gene age, but we showed that these phenomena do not dominate the coupling of codon usage to gene age.

The age dependence of codon usage was found to apply to all three representative organisms tested. Not only does this pattern remain as a general trend, but the dependence on age is in fact similar for the human, mouse, and fruit fly genomes. We conclude that the evolutionary history of an organism, over hundreds of million years, is strongly reflected in its codon usage.

## Methods

### Databases and Resources

Protein encoding gene sequences were obtained from the ENSEMBL database [24]. We included in our analysis only genes marked as 'known' and ignored genes that are annotated as 'novel'. In cases of alternative splicing variants, only a single splice variant was included. The numerous non-protein coding genes (including rRNA, tRNA, miRNA, snoRNA, etc.) were excluded. Genes encoding proteins of length shorter than 150 amino acids were also removed, since it is difficult to find statistically significant homologues for short proteins. A total of 17,274 human genes and 18,216 genes from mouse were included in the analysis.

For the analysis of the fly genome, we overcome the uneven full-genome sampling of the evolutionary tree from human to fly by taking advantage of the recent sequencing and annotation efforts of 12 species from the Drosophila genus [32]. The *D. melanogaster *proteome is based on the FlyBase database [38]. A total of 10,983 genes are included in the analysis.

For the human and mouse proteomes, homology was extracted from ENSEMBL using a predetermined list derived from a reciprocal BLAST identification scheme. These homologues are based on an exhaustive list of fully sequenced genomes (detailed in Figure 1) having high quality proteome annotations in a broad range of the evolutionary tree, from *H. sapiens *to *C. elegans *(Figure 1). Within the metazoa, we excluded the branch leading to distant phyla, including Placozoa, Porifera (sponges), and Cnidaria (corals and jellyfish) and focused on major model organisms of the animal kingdom. For the homologues from the perspective of the *D. melanogaster *genome (Table 2), we used the FlyBase database, in addition to ENSEMBL (including the *S. cerevisiae *genome), for obtaining the predetermined homologies of the genes [38].

Functional assignment for groups of genes is based on the enrichment of Gene Ontology (GO) annotations [37], with respect to the appearance of the GO annotation in the entire proteome.

### Age group assignments

The age of a gene was determined by the evolutionarily most distant genome containing an identified homologue to that gene. Thus, Age 1 denotes genes most specific to the organism being analyzed, and Age 9 (Age 8 for fly) includes genes found in the least common ancestor (LCA) of all analyzed species.

### Codon usage measurements

For each group of genes, codon usage frequencies were independently calculated for each of the amino acids. For each of the 18 degenerately encoded amino acids, the empirical frequencies of its corresponding codons were separately counted and normalized to sum to 1. The other two amino acids (tryptophan and methionine) each have a single codon and were not included in the analysis. Thus, each of the 59 redundant codons that account for these 18 amino acids were assigned a number between 0 and 1.

### Calculation of expected codon usage for a given GC content

We derived a method to calculate the codon usage expected for a given GC content, where this usage is most similar to a background codon usage. In our case, the background is the overall genomic codon usage. Formally, we seek the codon frequencies *f *that are closest to the background frequencies *B*, while constraining the GC content to a level of *G*:

where *D*_*KL *_denotes the Kullback-Leibler divergence, *n*^*j *^is the number of codons encoding amino acid *j*, *q*^*j *^is the relative frequency of amino acid *j*,  is the GC content of the *i*-th codon for the *j*-th amino acid, and  and  are the background and optimized frequencies of the *i*-th codon for the *j*-th amino acid, respectively.

We used Matlab to numerically solve this optimization problem. For each age group, the expected frequencies calculated by this method were compared to the observed frequencies using the χ^2 ^test for goodness of fit.

### Age dependence of codon usage

For all pairs of organisms analyzed, the similarity between the age-dependent responsiveness of their codon usages was calculated. In detail, for each genome analyzed, the deviation vector was defined as the difference of the codon usage vectors for 'old' and 'new' genes. Specifically, the 'new' set contains all genes in the Age 1 class (primate-specific for human, rodent and rabbit-specific for mouse, and melanogaster subgroup-specific for fly). The 'old' ones are those in all other age classes.

For example, we denote the usage vector of the 'new' human genes as:

where  denotes the frequency of the *i*-th codon (normalized by its respective amino acid). Next, we calculate the codon usage deviation vector between 'old' and 'new' human genes:

Finally, to compare human and mouse deviations, we calculated the angle between their respective vectors [*θ*(*human*, *mouse*)]:

The p-value of randomly finding two vectors with an incident angle whose cosine is at least as small as this was calculated as: [39].

## Abbreviations

GO: gene ontology; LCA: least common ancestor; MYA: millions of years; DKL: Kullback-Leibler divergence.

## Authors' contributions

YP conceived of the study, carried out the analysis, and drafted the manuscript. MF contributed to the analysis and to the writing of the manuscript. NL supported the statistical and analytical tests and edited the manuscript. ML led the research and contributed to the writing and the finalization of the manuscript.

## Supplementary Material

Additional file 1 **Age-dependent codon usage for human genes**. This file depicts the age-dependent codon usage for human genes, for each of the 18 degenerately coded amino acids (denoted by one letter codes). Click here for file

Additional file 2 **Age-dependent codon usage for mouse genes**. This file shows the age-dependent codon usage for mouse genes, for each of the 18 degenerately coded amino acids. Click here for file

Additional file 3 **Age-dependent codon usage for fly genes**. This file portrays the age-dependent codon usage for fly genes, for each of the 18 degenerately coded amino acids. Click here for file
